# Donor-Derived Cell-Free DNA at 1 Month after Kidney Transplantation Relates to HLA Class II Eplet Mismatch Load

**DOI:** 10.3390/biomedicines11102741

**Published:** 2023-10-10

**Authors:** Elena González-López, Javier Gonzalo Ocejo-Vinyals, Mónica Renuncio-García, Adriel Roa-Bautista, David San Segundo Arribas, Clara Escagedo, María del Mar García-Saiz, Rosalía Valero, Pilar García-Berbel, Juan Carlos Ruíz San Millán, Emilio Rodrigo

**Affiliations:** 1 Immunology Department, Immunopathology Group, Marqués de Valdecilla University Hospital-IDIVAL, University of Cantabria, 39008 Santander, Spain; elenagonzalez_lopez@hotmail.com (E.G.-L.); monica.renuncio@scsalud.es (M.R.-G.); aroaba17@gmail.com (A.R.-B.); david.sansegundo@scsalud.es (D.S.S.A.); 2 Immunology Department, Infectious Diseases and Clinical Microbiology Group, Marqués de Valdecilla University Hospital-IDIVAL, University of Cantabria, 39008 Santander, Spain; javiergonzalo.ocejo@scsalud.es; 3 Nephrology Department, Immunopathology Group, Marqués de Valdecilla University Hospital-IDIVAL, University of Cantabria, 39008 Santander, Spain; clara.escagedo@scsalud.es (C.E.); rosalia.valero@scsalud.es (R.V.); juancarlos.ruiz@scsalud.es (J.C.R.S.M.); 4 Clinical Pharmacology Department, Marqués de Valdecilla University Hospital-IDIVAL, University of Cantabria, 39008 Santander, Spain; mmar.garcia@scsalud.es; 5 Pathological Anatomy Department, Marqués de Valdecilla University Hospital-IDIVAL, University of Cantabria, 39008 Santander, Spain; pgberbel@gmail.com

**Keywords:** kidney transplant, dd-cfDNA, graft damage, HLA eplet mismatch load

## Abstract

Kidney transplantation is the preferred therapeutic option for end-stage renal disease; however, the alloimmune response is still the leading cause of renal allograft failure. To better identify immunologic disparities in order to evaluate HLA compatibility between the donor and the recipient, the concept of eplet load has arisen. Regular kidney function monitoring is essential for the accurate and timely diagnosis of allograft rejection and the appropriate treatment. Donor-derived cell-free DNA (dd-cfDNA) has been proposed as a potential biomarker of acute rejection and graft failure in kidney transplantation. The proportion of plasma dd-cfDNA was determined in forty-two kidney patients at 1 month after transplantation. A total of eleven (26.2%) patients had a dd-cfDNA proportion of ≥1.0%. The only pretransplant variable related to dd-cfDNA > 1.0% was the HLA class II eplet mismatch load, mainly the HLA-DQB1 eplet mismatch load. Furthermore, dd-cfDNA was able to discriminate the patients with antibody-mediated rejection (AbMR) (AUC 87.3%), acute rejection (AUC 78.2%), and troubled graft (AUC 81.4%). Increased dd-cfDNA levels were associated with kidney allograft deterioration, particularly rejection, as well as a greater HLA class II eplet mismatch load. Consequently, combining dd-cfDNA determination and HLA eplet mismatch load calculation should improve the assessment of the risk of short- and long-term allograft damage.

## 1. Introduction

Kidney transplantation is the preferred therapeutic option for end-stage renal disease. In this research field, a marked improvement in one-year graft survival rates has been shown [1], although alloimmune response is still the leading cause of renal allograft failure [2]. The most important non-self-antigens implicated in the alloimmune response are the human leukocyte antigens (HLA) [3]. In kidney transplantation, HLA mismatch between the donor and the recipient has been associated with early acute rejection and poor graft outcome [4], leading to T-cell-mediated rejection (TCMR) and antibody-mediated rejection (AbMR) [5,6].

The concept of eplets has emerged as a novel method to fine-tune the HLA risk and better identify immunologic disparities in a more detailed way in order to evaluate HLA compatibility between the donor and the recipient, which may lower the risk of allograft rejection [7,8,9,10]. Several studies have demonstrated a relationship between donor–recipient eplet disparity and graft outcome in renal transplantation [11,12,13].

Effective immunosuppressive treatments are another important strategy for ensuring long-term transplant survival [14]. Nonetheless, maintaining an appropriate immunosuppressive balance is crucial in order to avoid severe side effects, such as infection and cancer [15]. Consequently, regular kidney function monitoring is essential for the accurate and timely diagnosis of allograft rejection and the appropriate treatment [16].

Several biomarkers, including serum creatinine levels and estimated glomerular filtration rate (eGFR), are used to monitor graft function and rejection, but their capacity to identify early damage is limited and they may not discriminate acute from chronic graft damage [17,18]. The gold standard for diagnosing rejection and graft decline is the histologic analysis of the allograft biopsy [19]. However, biopsies are unsuitable for routine monitoring since they are invasive, costly, and risk consequences.

As a result, non-invasive and new indicators of early and late graft failure in renal transplantation are required to enhance complication management and increase patient and graft survival [20,21]. To achieve this, donor-derived cell-free DNA (dd-cfDNA) has been proposed as a potential biomarker of acute rejection and graft failure in kidney transplants [21,22]. With a size of 150–180 base pairs, the majority of circulating cfDNA is released into the bloodstream via cell apoptosis [23], and its levels are altered in chronic inflammation, malignancies, and injury, which can be clinically useful [16,24]. Several studies have shown that dd-cfDNA levels rise immediately after a kidney transplant and subsequently decline within 10 days [25]. Moreover, higher initial levels of circulating dd-cfDNA have been observed in deceased donors, rejection, or graft failure and in situations of insufficient immunosuppression [26].

## 2. Materials and Methods

### 2.1. Study Design and Patients’ Information

We conducted this prospective study in accordance with the principles outlined in the Declaration of Helsinki and with approval from our institution’s Regional Ethics Committee (reference number: PI20/01710; 22 December 2020). We enrolled a total of 42 consecutive recipients of kidney transplants from deceased donors, spanning from January 2021 to April 2022. All the participants provided written consent before undergoing kidney transplantation. We excluded recipients who had received non-kidney solid organ transplants, those from non-controlled cardiac death donations, individuals with preformed donor-specific antibodies, and those with a panel-reactive antibody level exceeding 98%.

We gathered relevant information regarding the recipients, donors, and transplant characteristics. Biopsy confirmation was required for all acute rejection episodes. Indication biopsies were performed when creatinine levels increased by 25% or more compared to the previous value, or when there was persistent proteinuria exceeding 1 g per day. We defined a “troubled” graft as one undergoing acute rejection or other issues, such as obstruction or graft infection. Maintenance immunosuppressive therapy consisted of twice-daily doses of tacrolimus, mycophenolate mofetil, and prednisone. Mycophenolate mofetil was started at 500 mg twice daily, whereas the initial tacrolimus dose was 0.1 mg/kg twice daily. The target tacrolimus blood levels at our institution for the first 3 months ranged from 8 to 12 ng/mL. Recipients of organs from expanded criteria donors and those at risk of delayed graft function received basiliximab induction therapy. Thymoglobulin was used as induction therapy for patients at a higher risk of rejection due to hypersensitization or previous graft loss caused by acute rejection. All patients received trimethoprim–sulfamethoxazole prophylaxis for 6 months post-transplant, and valganciclovir was given for 3 months to CMV IgG-negative recipients of a CMV IgG-positive donor and in patients receiving thymoglobulin induction.

### 2.2. dd-cfDNA Determination

Peripheral blood samples were collected from kidney transplant recipients at 1 month post-transplant into Streck Cell-Free DNA BCT tubes. Then, after centrifugation steps of 1,600× *g* for 20 min and 16,000× *g* for 10 min, plasma was obtained. cfDNA was extracted using the QIAamp Circulating Nucleic Acid Kit (Qiagen, Redwood City, CA, USA) and to determine the relative amount of dd-cfDNA, a targeted NGS assay employing 202 single nucleotide polymorphisms (SNPs) was then utilized (AlloSure^®^, CareDx, Inc., Brisbane, CA, USA) (Table S1). First, a multiplexed polymerase chain reaction (PCR) was performed with primers to amplify the targeted SNPs and the joining of indexes to identify the samples, as well as adapters required for the sequencing step. The amplified product was then pooled and purified in preparation for sequencing. The result of PCR was sequenced on Illumina (Illumina, Inc. Brisbane, CA, USA) MiSeq equipment. Finally, dd-cfDNA% was calculated using CareDx AlloSeq cfDNA software 1.0 (“Allosoft”) by using informative SNPs between donor and recipient. The kit amplified 202 SNPs that were consciously selected along the 22 autosomal chromosomes. After reading the amplified SNPs using NGS, the number of reads for each nucleotide within each of the SNPs could be identified, making it feasible to identify the cfDNA of the donor and the recipient. Because only post-transplant samples were used, and there was no prior donor–recipient genotyping, the program only used homozygous informative SNPs to complete the analysis. When the percentage of two-nucleotide readings at an SNP site was outside the range of 30–70%, a homozygous SNP was regarded as informative. For each SNP, the nucleotide with the highest number of reads related to the recipient, while the one with the lowest proportion corresponded to the donor (e.g., if there are 2% of “A” reads and 98% of “G” reads, the donor is AA and the recipient is GG for that SNP, and this was used to calculate the % dd-cfDNA). Finally, the computer calculated an average of the percentage of nucleotide readings from the informative SNPs that correspond to the donor. Furthermore, AlloSoft considers several quality parameters to ensure the reliability of the calculated results, such as the uniformity of readings obtained between different SNPs, the possibility of contamination of the sample through the detection of outliers and total readings. The reporting of % of dd-cfDNA in the literature allows for the assumption that the free DNA of a lower quantity comes from the donor in the post-transplant sample. It can be shown that the percentage of dd-cfDNA in lung [27], kidney [16], and heart [28] transplants is always lower than the percentage of free DNA in the patient. A cutoff of ≥1.0% was used, in accordance with the manufacturer’s instructions, as an anomalous dd-cfDNA result.

### 2.3. Immunosuppressive Drugs Monitoring

We quantified the concentration of mycophenolic acid (MPA) in human plasma (mg/L) using a homogeneous enzyme immunoassay (Emit 2000 Mycophenolic Acid Assay; Siemens, Saint Paul, MN, USA) at the one-month mark. Whole blood concentrations (µg/L) of tacrolimus were determined using chemiluminescent microparticle immunoassay (CMIA; Abbott Laboratories, Abbott Park, IL, USA) on the Architect iSystem. We collected tacrolimus levels up to day 30. We assessed the variability of tacrolimus blood levels using the coefficient of variation (CV), calculated as CV (%) = (σ/μ) × 100, where σ represents the standard deviation, and μ denotes the mean tacrolimus concentration of all available samples [26]. We determined the percentage of time within the therapeutic range (8 to 12 ng/mL) and above 12 ng/mL using the Rosendaal method [27]. Tacrolimus C/D (concentration-to-dose) ratios were calculated at the one-month and three-month marks, with fast metabolizers defined as those having a tacrolimus level–dose ratio below 1.05 [28]. “Any tacrolimus level < 5 ng/mL at month 1” and “any tacrolimus level < 6 ng/mL at month 1” were defined if the patients had at least one trough tacrolimus blood level below the threshold of 5 ng/mL or 6 ng/mL at any time up to month 1.

### 2.4. Eplet Mismatch Examination

Antigen HLA and eplet mismatch load were calculated to establish donor and recipient compatibility. The HLAMatchmaker 3.1 software (available at http://www.epitopes.net/downloads.html accessed on 15 April 2023) was used to assess eplet matching.

### 2.5. Statistical Analysis

The median and interquartile range (IQR) were used to express continuous variables, and relative frequencies were used to characterize categorical variables. To compare continuous variables among dichotomous data, the Mann–Whitney U test was used, and the chi-square test was used to investigate the relationship between two qualitative variables. The ability of dd-cfDNA% to distinguish between rejection and troubled graft was examined via receiver operating characteristic (ROC) curves. Statistical significance was defined as a *p*-value less than 0.05. Statistical analyses were performed with SPSS, version 15.0 (SPSS, Inc., Chicago, IL, USA).

## 3. Results

### 3.1. Patient Characteristics

The main patient characteristics are shown in Table 1. The median dd-cfDNA value in kidney transplant recipients was 0.61, with an interquartile range from 0.34 to 1.06. A total of 11 patients (26.2%) had a percentage of dd-cfDNA ≥ 1.0%. Differences in variables between patients with dd-cfDNA below and above 1.0% are shown in Table 1. The only pretransplant variable related to dd-cfDNA percentage was HLA class II eplet mismatch load, predominantly the HLA-DQB1 eplet mismatch load (*p* = 0.022 and *p* = 0.041, respectively) (Table 2). Interestingly, neither antigen HLA mismatch number nor HLA class I eplet mismatch load was associated with dd-cfDNA. Moreover, immunosuppressive exposure was not related to dd-cfDNA.

### 3.2. dd-cfDNA% and Troubled Graft Patients

As presented in Table 1, patients with acute rejection, AbMR, and troubled grafts in the first month post-transplant had significantly higher levels of dd-cfDNA%. Seven patients developed acute rejection within the first month; the median time was 14 days after transplantation (range 9–30 days). Among the 11 patients with a dd-cfDNA ≥ 1.0%, 4 patients developed acute rejection, and 3 of them were suffering different problems that could justify the elevation of dd-cfDNA% (2 patients needed a nephrostomy tube due to graft hydronephrosis, 1 of them developed a urinary sepsis due to Klebsiella, and 1 required a bladder catheter due to urinary obstruction). Moreover, 4 patients had a dd-cfDNA ≥ 1.0% without a clear cause: 1 patient developed cytomegalovirus infection in the first month, and 1 patient needed a percutaneous tube to drain a peri-graft collection without hydronephrosis. Among 5 patients with AbMR, only 1 of them had a dd-cfDNA < 1.0% (0.69%), but this sample was obtained after finishing AbMR therapy with plasmapheresis plus intravenous immunoglobulins. Two patients with isolated borderline TCMR had dd-cfDNA values below 1.0% (0.74 and 0.53). 

### 3.3. dd-cfDNA% in AbMR Patients

**Figure 1:**
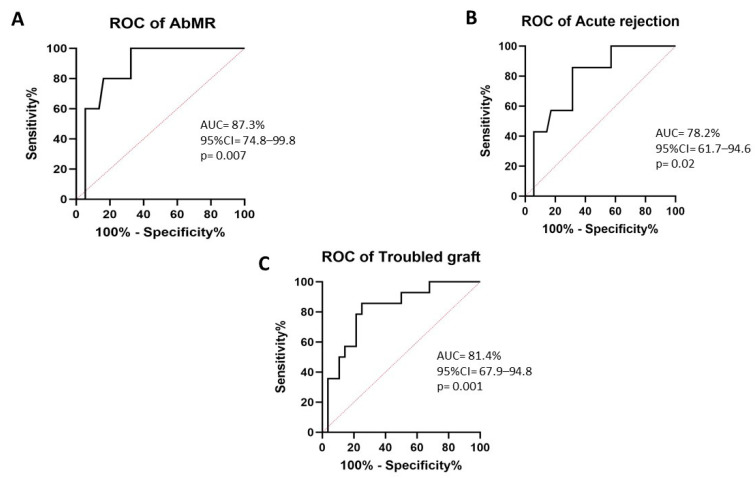
AUC–ROC curve of dd-cfDNA% for discriminating AbMR (**A**), acute rejection (**B**), and troubled graft (**C**). Abbreviations: AbMR—antibody-mediated rejection; dd-cfDNA—donor-derived cell-free DNA.

**Figure 2:**
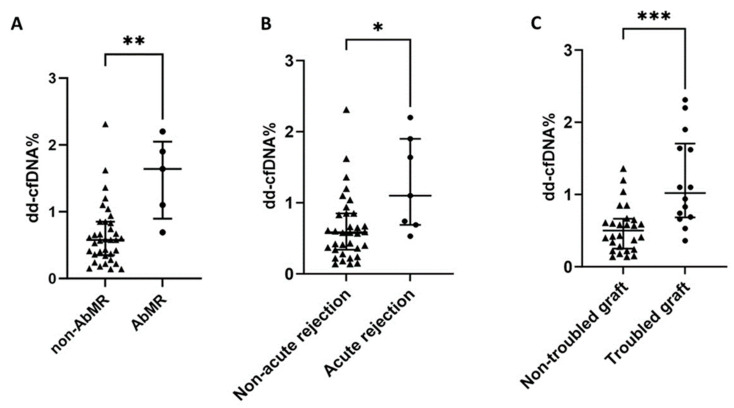
The median and interquartile range of dd-cfDNA% in patients with and without AbMR (**A**), acute rejection (**B**), and troubled graft (**C**). Abbreviations: AbMR—antibody-mediated rejection; dd-cfDNA—donor-derived cell-free DNA. * *p* < 0.05; ** *p* < 0.01; *** *p* < 0.001.

dd-cfDNA was able to discriminate those patients with AbMR (AUC-ROC 87.3%, 95%CI 74.8–99.8, *p* = 0.007) (Figure 1A), with acute rejection (AUC-ROC 78.2, 95%CI 61.7–94.6, *p* = 0.020) (Figure 1B), and with troubled kidney graft (AUC-ROC 81.4, 95%CI 67.9–94.8, *p* = 0.001) (Figure 1C). Patients with AbMR (1.64, IQR 1.15 vs. 0.58, IQR 0.50, *p* = 0.004) (Figure 2A), acute rejection (1.10, IQR 1.21 vs. 0.58, IQR 0.51, *p* = 0.017) (Figure 2B), and troubled graft (1.02, IQR 1.02 vs. 0.50, IQR 0.42, *p* = 0.001) (Figure 2C) had significantly higher levels of dd-cfDNA. Moreover, patients with a dd-cfDNA ≥ 1.0% had a significantly higher risk of AbMR (HR 27.03, 95%CI 1.27–577.01, *p* = 0.035), independent of renal function. Notably, AbMR did not relate to a higher eplet mismatch load.

## 4. Discussion

Regular graft function monitoring in kidney transplant recipients is critical for the accurate detection of graft deterioration and starting appropriate therapy to avert graft loss. dd-cfDNA has emerged as a new and non-invasive biomarker for graft injury in kidney transplants, with evidence that it overcomes the limitations of traditional approaches. It has been demonstrated that a dd-cfDNA level of >1.0% is associated with active rejection (AbMR or TCMR), and that those patients with levels of dd-cfDNA of 1.0% or below do not show active rejection [16]. Accordingly, when using 1% as a cutoff point in kidney transplants, the negative predictive value of dd-cfDNA is 84% for active rejection and 96% for antibody-mediated rejection [16]. The median dd-cfDNA levels found in different kidney transplant groups with rejection ranged from 1.0% to 3.0% [16,24,29,30]. Our results show that the majority of the patients who developed rejection (AbMR) at 1-month post-transplant (36.4%) had levels of dd-cfDNA ≥ 1.0%.

Similarly, we demonstrated that dd-cfDNA levels ≥ 1.0% can differentiate patients with acute rejection (AUC–ROC 78.2%, 95%CI 61.7–94.6, *p* = 0.020), especially AbMR (AUC–ROC 87.3%, 95%CI 74.8–99.8, *p* = 0.007), from those without rejection. This ability to discriminate those patients with rejection has been proved in a previously reported study. Sigdel et al. [24] and Zhang et al. [30] reported that the relative dd-cfDNA determination could differentiate between patients with and without acute rejection (AUC–ROC of 87% and 90%, respectively), whereas Gielis et al. [31] reported an AUC–ROC of 64% with a cutoff of 0.88%. There are two approaches for quantifying dd-cfDNA: relative and absolute [32]. Whitlam et al. [33] demonstrated that absolute dd-cdDNA (cp/mL) determination has a 91% AUC–ROC in the diagnosis of AbMR. Furthermore, they assessed the sensitivity and specificity for relative and absolute dd-cfDNA measurements, revealing 75% and 79% specificity, respectively, and the same sensitivity (85%) for the two approaches. The researchers concluded that both might be equally useful in detecting rejection in kidney transplant recipients.

Previous studies have demonstrated that dd-cfDNA is not effective in distinguishing TCMR from non-rejection or stable patients with lower levels than patients with AbMR [34], and similar findings have been reported in heart [35] and lung [36] transplantation. Because of the small number of pure TCMR in our data limits, we could not perform a non-AbMR-specific analysis. 

In this study, dd-cfDNA% was assessed one month after transplant. The long-term effects of solid organ transplantation are still far from desirable. Studies have examined the potential of serial dd-cfDNA for long-term monitoring in kidney transplant recipients with acute rejection and intravenous steroid therapy. Shen et al. [37] observed a decline in dd-cfDNA% at 3 days and at 1, 3, and 6 months, correlating with estimated glomerular filtration, which indicated a response to rejection treatment. In addition, when combined with other clinically relevant findings (infections, malignancies), dd-cfDNA long-term monitoring might aid in the adaptation of immunosuppressive therapy [38].

Furthermore, we investigated the ability of dd-cfDNA to distinguish various sources of allograft damage or loss of function. Patients were classified into two groups: troubled graft, if the graft was suffering acute rejection or any other problem such as obstruction or graft infection, and non-troubled graft. dd-cfDNA levels of more than 1.0% were able to distinguish between these two groups (AUC–ROC 81.4%, 95%CI 67.9–94.8, *p* = 0.001). In line with this, Goussouss et al. [39] reported seven cases in which transplant infection was linked with an increase in plasma dd-cfDNA. In terms of obstruction, ureteral blockage occurs in 2–10% of renal transplant patients within the first few weeks or the first year [40], and to the best of our knowledge, this is the first study to incorporate obstruction as a post-renal transplantation complication in the analysis of dd-cfDNA.

In histocompatibility laboratories, antigen HLA mismatch is a common examination factor in kidney transplant candidates. However, the idea of epitope “load” has recently emerged as a novel technique to better characterize donor–recipient immunologic compatibility in solid organ transplantation. Matching recipients and donors according to the level of epitopes, as compared with traditional antigen HLA typing, may improve risk prediction for the development of donor-specific antibodies implicated in AbMR [41]. Thus, we found that patients with dd-cfDNA levels of more than 1.0% had a higher HLA class II eplet mismatch load (46 vs. 21, *p* = 0.022). Moreover, in recent years, the importance of antigen HLA-DQ matching for transplant outcome has been highlighted [42]. Several studies have shown that HLA-DQ eplet mismatch load is associated with an increased risk of developing de novo donor-specific antibodies, which are implicated in kidney transplant rejection, graft function decline, and graft failure [43,44,45], which may influence dd-cfDNA levels. In addition, urinary CXCL10, a non-invasive biomarker associated with inflammation and graft survival, has been linked to HLA-DBQ1 eplet mismatch load [46]. In this context, we found that elevated HLA-DQB1 eplet mismatch load was associated with higher levels of dd-cfDNA (*p* = 0.041). HLA matching between potential donors and recipients is assessed in kidney allocation by comparing their HLA antigens in order to have a successful transplant outcome, limiting the possibility of sensitization and subsequent rejection [47]. Unexpectedly, we found no relationship between conventional antigen HLA class I and class II mismatch and the percentage of dd-cfDNA. To our knowledge, this work is the first to show an association between the presence of HLA class II eplet mismatch with elevated dd-cfDNA levels (≥1.0%).

The main limitation of this single-center study was the sample size; we could only assess dd-cfDNA% in forty-two kidney transplant recipients one month after transplant. Because of the small sample size, the number of events, especially TCMR, was limited, restricting the capacity to demonstrate the relationship between dd-cfDNA and this type of rejection. Our study, on the other hand, has certain advantages. We conducted a prospective analysis on a cohort of kidney transplant patients who were followed in a single center, and all rejections were biopsy-proven. Furthermore, we conducted an in-depth study of the eplet mismatch load, which has not previously been associated with the percentage of dd-cfDNA.

## 5. Conclusions

Our findings support the value of measuring dd-cfDNA to detect alloimmune damage, particularly AbMR, in kidney transplantation. Only recipients with a high eplet mismatch load with their donors and an elevated percentage of dd-cfDNA were at risk of acute rejection and AbMR. Furthermore, an optimum assessment of the risk of short- and long-term allograft injury should involve the quantification of both the eplet mismatch load and post-transplant dd-cfDNA percentage.

## Figures and Tables

**Table 1:** Main patient characteristics.

Variable	Total (*n* = 42)	dd-cfDNA ≥ 1.0% (*n* = 11)	dd-cfDNA < 1.0% (*n* = 31)	*p*
Recipient age (years)	59.0 [47.8–67.5]	60.0 [48.0–73.0]	59.0 [47.0–66.0]	0.572
Recipient gender (male)	76.2%	63.6%	80.6%	0.255
Diabetic nephropathy	23.8%	18.2%	25.8%	0.610
Time in renal replacement therapy (months)	13.5 [3.9–38.7]	24.5 [10.5–108.7]	10.7 [0.0–32.6]	0.062
Retransplant	19.0%	36.4%	12.9%	0.089
Preemptive transplantation	21.4%	18.2%	22.6%	0.760
Donor age (years)	58.5 [47.0–65.0]	61.0 [47.0–65.0]	58.0 [47.0–65.0]	0.910
Cold ischemia time (hours)	21.5 [17.8–24.0]	20.0 [18.0–24.0]	22.0 [17.0–24.0]	0.822
Induction	66.7%	72.7%	64.5%	0.620
1-month acute rejection	16.7%	36.4%	9.7%	0.041
1-month AbMR	11.9%	36.4%	3.2%	0.004
1-month troubled graft	33.3%	63.6%	22.6%	0.013
First year acute rejection (RAPreM12)	21.4%	36.4%	16.1%	0.160
First month eGFR (mL/min/1.73 m^2^)	49.5 [40.5–70.0]	43.0 [28.0–54.0]	51.0 [42.0–72.0]	0.138
First month albuminuria (mg/g)	55.0 [23.5–150.5]	57.5 [34.5–485.8]	53.0 [22.5–135.5]	0.524
1-year eGFR (mL/min/1.73 m^2^)	50.0 [38.0–68.0]	45.0 [38.0–49.0]	50.0 [38.0–71.8]	0.514
dd-cfDNA (%)	0.61 [0.34–1.06]	-	-	0.396
**Data of immunosuppressive therapy**				
TTR 8–12 at month 1 (%)	45.7 [29.3–61.3]	50.9 [37.0–60.4]	45.5 [25.0–61.4]	0.396
TTR > 12 at month 1 (%)	47.3 [34.0–65.2]	49.1 [39.6–63.0]	44.8 [33.6–75.0]	1.000
Mean tacrolimus trough level throughout month 1 (ng/mL)	12.5 [11.3–14.3]	12.5 [11.4–14.3]	12.3 [11.2–14.2]	0.822
Tacrolimus trough level at month 1 (ng/mL)	12.1 [10.0–14.0]	13.0 [10.0–15.0]	12.0 [9.0–14.0]	0.233
Mycophenolic acid trough level at month 1 (ng/mL)	2.0 [1.0–3.0]	2.0 [1.8–4.0]	2.0 [1.0–3.0]	0.569
Any tacrolimus level < 5 at month 1	7.1%	0.0%	9.7%	0.284
Any tacrolimus level < 6 at month 1	11.9%	0.0%	16.1%	0.156
Coefficient of variability at month 1 (%)	28.1 [19.4–34.2]	21.8 [16.1–61.5]	28.6 [19.8–37.2]	0.257
Tacrolimus trough level/dose at month 1	1.7 [1.3–2.5]	1.8 [1.3–2.5]	1.7 [1.1–2.7]	0.778
Fast tacrolimus metabolizers	11.9%	9.1%	12.9%	0.737

Mann–Whitney U and Chi-square tests. Abbreviations: TTR—time in therapeutic range.

**Table 2:** HLA mismatches at the antigen and eplet levels between donor and recipient.

Variable	Total (*n* = 42)	dd-cfDNA ≥ 1.0% (*n* = 11)	dd-cfDNA < 1.0% (*n* = 31)	*p*
Antigen HLA class I and II mismatches	7.5 [6.0–9.0]	8.0 [7.0–9.0]	7.0 [5.0–9.0]	0.233
All HLA class I eplet mismatch load	18.0 [13.0–22.0]	20.0 [16.0–22.0]	16.0 [13.0–22.0]	0.553
All HLA class II eplet mismatch load	23.5 [14.8–51.5]	46.0 [22.0–69.0]	21.0 [11.0–39.0]	0.022
HLA-DRB1 eplet mismatch load	9.0 [4.0–14.0]	10.0 [7.0–14.0]	9.0 [3.0–13.0]	0.245
HLA-DQB1 eplet mismatch load	7.0 [2.8–9.3]	9.0 [7.0–11.0]	7.0 [2.0–9.0]	0.041
HLA-DQA1 eplet mismatch load	1.0 [0.0–5.0]	3.0 [0.0–5.0]	1.0 [0.0–4.0]	0.445

Mann–Whitney U and Chi-square tests.

## Data Availability

The data presented in this study are available upon request from the corresponding author.

## Supplementary Materials

The following supporting information can be downloaded at: https://www.mdpi.com/article/10.3390/biomedicines11102741/s1, Table S1: Data from the SNPs studied.
