# Supplementary material for: Donor-Derived Cell-Free DNA at 1 Month after Kidney Transplantation Relates to HLA Class II Eplet Mismatch Load
Source: Biomedicines. 2023 Oct 10;11(10):2741. doi: 10.3390/biomedicines11102741 (PMC10604614; doi:10.3390/biomedicines11102741)
Supplement: Supplementary file 1 [file biomedicines-11-02741-s001.zip › biomedicines-2589025-supplementary.pdf]

Supplementary Table S1. Data from the SNPs studied.

AlloSeq name	Chromosome	Chr. Location	Unique Gene
SNP137	**1**	1p36.31	CAMTA1: Intron Variant
SNP119	1	1p31.2	LOC105378787: Intron Variant
SNP030	1	1p21.1	None
SNP071	1	1p13.3	None
SNP057	1	1p13.2	NGF-AS1: Intron Variant LOC11226823: Intron Variant
SNP179	1	1p12	None
SNP125	1	1q41	None
SNP048	1	1q43	None
SNP163	2	2p25.3	SNTG2: Intron Variant
SNP105	2	2p24.1	LINC01808: Intron Variant
SNP093	2	2p24.1	None
SNP054	2	2p23.3	None
SNP175	2	2p22.2	CRIM1: Intron Variant
SNP003	2	2p22.1	LOC105374497: Non Coding Transcript Variant
SNP141	2	2p21	LINC01913: Intron Variant
SNP046	2	2p16.3	None
SNP088	2	2p16.2	LOC105369165: Intron Variant
SNP007	2	2p14	None
SNP166	2	2q12.1	TMEM182: Intron Variant
SNP097	2	2q12.2	None
SNP117	2	2q14.1	POLR1B: Intron Variant; LOC105373562: 2KB Upstream Variant
SNP189	2	2q21.2	LOC105373628: Intron Variant
SNP169	2	2q22.3	LINC01412: Intron Variant
SNP089	2	2q22.3	None
SNP095	2	2q23.3	None
SNP151	2	2q31.1	LRP2: Intron Variant

SNP020	2	2q32.1	FRZB: Intron Variant
SNP184	2	2q33.3	None
SNP038	2	2q35	LOC101928278: Intron Variant
SNP158	2	2q37.3	HDAC4: Intron Variant
SNP170	3	3p26.1	ITPR1: Intron Variant
SNP138	3	3p24.1	None
SNP022	3	3p22.3	None
SNP149	3	3p22.3	STAC: Intron Variant
SNP181	3	3p22.1	None
SNP024	3	3P14.2	LOC105377110: Intron Variant
SNP063	3	3p13	None
SNP019	3	3q11.2	None
SNP082	3	3q12.1	TMEM30CP: Intron Variant
SNP053	3	3q13.2	None
SNP182	3	3q13.31	CCDC191: Intron Variant; LOC105374048: Intron Variant
SNP161	3	3q21.3	None
SNP027	3	3q22.1	LOC105374107: Intron Variant; LOC107986023: Intron Variant
SNP034	3	3q22.1	TMEM108: Intron Variant; LOC101927432: Non Coding Transcript Variant
SNP043	3	3q24	None
SNP111	3	3q24	None
SNP193	3	3q25.1	ERICH6: Intron Variant
SNP144	3	3q25.1	CLRN1: Intron Variant
SNP078	3	3q25.2	MBNL1: Intron Variant
SNP115	3	3q25.2	None
SNP011	3	3q26.31	SPATA16: Intron Variant
SNP068	3	3q27.3	LOC105374258: Intron Variant
SNP064	3	3q28	None
SNP006	4	4p16.2	STK32B: Intron Variant
SNP178	4	4p15.31	None
SNP150	4	4p15.1	PCDH7: Intron Variant

SNP050	4	4p14	TBC1D1: Intron Variant
SNP192	4	4q12	LOC105377663: Intron Variant
SNP152	4	4q26	None
SNP195	4	4q28.3	None
SNP047	4	4q31.21	None
SNP155	4	4q31.3	None
SNP005	4	4q32.2	None
SNP124	4	4q35.1	None
SNP001	4	4q35.1	LINC02427: Intron Variant
SNP153	5	5p13.3	None
SNP132	5	5p13.3	None
SNP147	5	5p13.3	ADAMTS12: Intron Variant
SNP010	5	5q11.2	None
SNP028	5	5q11.2	None
SNP200	5	5q13.1	None
SNP118	5	5q14.1	LOC101929154: Intron Variant
SNP172	5	5q21.1	None
SNP094	5	5q21.1	None
SNP086	5	5q21.3	None
SNP031	5	5q22.3	None
SNP174	5	5q23.1	None
SNP136	5	5q31.3	HBEGF: 3 Prime UTR Variant
SNP069	5	5q33.1	GLRA1: Intron Variant
SNP127	5	5q34	None
SNP101	5	5q34	None
SNP025	6	6p25.1	LY86-AS1: Intron Variant
SNP106	6	6p21.2	C6orf89: Intron Variant; CPNE5: Intron Variant
SNP081	6	6p21.2	GLP1R: Synonymous Variant; LOC105375046: 2KB Upstream Variant
SNP021	6	6q13	None
SNP202	6	6q13	COL12A1: Intron Variant
SNP121	6	6q14.1	LINC02542: Intron Variant
SNP061	6	6q22.1	LINC02534: Intron Variant
SNP176	6	6q22.31	None
SNP044	6	6q22.31	NKAIN2: Intron Variant

SNP065	6	6q22.33	C6orf58: Intron Variant
SNP076	6	6q22.33	LAMA2: Intron Variant
SNP074	6	6q22.33	None
SNP037	6	6q23.3	PDE7B: Intron Variant; LOC101928373: Intron Variant
SNP080	6	6q25.3	None
SNP187	6	6q25.3	ZDHHC14: Intron Variant
SNP026	6	6q25.3	None
SNP059	6	6q26	PRKN: Intron Variant
SNP002	7	7p22.3	None
SNP055	7	7p21.1	None
SNP173	7	7p14.3	CHN2: Intron Variant
SNP131	7	7p12.3	None
SNP113	7	7q22.2	LHFPL3: Intron Variant
SNP032	7	7q31.2	WNT2: Intron Variant; LOC105375467: Intron Variant
SNP004	7	7q35	TPK1: Intron Variant
SNP162	7	7q36.3	LMBR1: Intron Variant
SNP085	8	8p21.2	None
SNP133	8	8p21.2	None
SNP129	8	8p21.1	SCARA3: Intron Variant
SNP201	8	8p21.1	ELP3: Intron Variant
SNP107	8	8p11.22	LOC105379384: Intron Variant
SNP142	8	8q12.1	None
SNP096	8	8q21.11	MIR5681A: 2KB Upstream Variant
SNP188	8	8q23.1	None
SNP012	8	8q24.21	CCDC26: Intron Variant
SNP087	9	9p24.3	LOC105375951: Intron Variant
SNP160	9	9p22.3	None
SNP199	9	9p21.3	LOC101929563: Intron Variant
SNP009	9	9p21.1	None
SNP072	9	9q21.13	GDA: Intron Variant
SNP100	9	9q21.13	LOC101927329: Intron Variant

SNP023	9	9q21.13	LOC105376091: Intron Variant
SNP099	9	9q31.3	None
SNP042	9	9q32	HSDL2: Intron Variant
SNP186	9	9q32	TMEM268: Intron Variant
SNP098	9	9q33.3	SCAI: Intron Variant
SNP108	9	9q34.11	None
SNP029	10	10p14	None
SNP177	10	10p13	CAMK1D: Intron Variant
SNP180	10	10q22.1	CDH23: Intron Variant
SNP056	10	10q23.31	LOC105378430: Intron Variant
SNP077	10	10q24.1	ZDHHC16: Intron Variant
SNP014	10	10q25.3	AFAP1L2: Intron Variant
SNP197	10	10q26.3	None
SNP139	11	11p15.1	NELL1: Intron Variant
SNP154	11	11p13	LDLRAD3: Intron Variant
SNP015	11	11q13.5	None
SNP143	11	11q14.1	TENM4: Intron Variant
SNP164	11	11q14.2	CCDC83: Intron Variant
SNP191	11	11q14.2	LOC105369421: Intron Variant
SNP122	11	11q23.3	CADM1: Intron Variant
SNP062	11	11q23.3	DSCAML1: Intron Variant
SNP049	11	11q24.1	MIR100HG: Intron Variant
SNP120	11	11q24.3	FLI1: Intron Variant; SENCR: 2KB Upstream Variant
SNP103	12	12p13.33	ADIPOR2: Intron Variant
SNP110	12	12p13.32	TIGAR: 2KB Upstream Variant
SNP092	12	12q14.2	None
SNP060	12	12q22	None
SNP114	12	12q23.3	None
SNP165	12	12q24.22	None
SNP039	12	12q24.23	KSR2: Intron Variant
SNP070	12	12q24.31	MLXIP: Intron Variant

SNP091	12	12q24.32	LINC02372: Non Coding Transcript Variant
SNP018	13	13q13.3	None
SNP016	13	13q21.2	None
SNP126	13	13q21.32	None
SNP051	13	13q21.33	None
SNP075	13	13q31.3	None
SNP035	13	13q33.1	NALCN: Intron Variant
SNP135	13	13q33.3	EFNB2: Intron Variant
SNP066	14	14q11.2	ABHD4: Intron Variant
SNP104	14	14q13.1	NPAS3: Intron Variant
SNP109	14	14q13.1	EGLN3: Intron Variant
SNP013	14	14q13.2	LOC105370452: Intron Variant
SNP052	14	14q24.3	None
SNP041	14	14q32.11	EFCAB11: Intron Variant
SNP090	14	14q32.13	None
SNP123	14	14q32.2	-
SNP058	15	15q14	None
SNP102	15	15q14	None
SNP033	15	15q21.2	USP8: Intron Variant
SNP008	15	15q22.2	RORA: Intron Variant; RORA-AS1: Intron Variant
SNP196	15	15q26.1	None
SNP156	15	15q26.1	None
SNP036	16	16q12.1	ZNF423: Intron Variant
SNP083	16	16q21	None
SNP017	17	17p13.3	NXN: Intron Variant
SNP145	17	17p12	HS3ST3A1: Intron Variant
SNP112	17	17p12	COX10: Intron Variant
SNP140	17	17q12	None
SNP168	17	17q23.2	MARCHF10-DT: Intron Variant
SNP067	18	18p11.31	None
SNP159	18	18p11.22	None
SNP116	18	18p11.22	None
SNP040	18	18q11.2	None
SNP157	18	18q21.1	ZBTB7C: Intron Variant
SNP146	18	18q21.31	NEDD4L: Intron Variant

SNP198	19	19p13.2	-
SNP079	19	19q12	None
SNP128	19	19q12	TSHZ3: Intron Variant
SNP185	19	19q13.31	IRGQ: 3 Prime UTR Variant
SNP171	20	20p12.1	MACROD2: Intron Variant
SNP183	20	20p11.22	None
SNP134	20	20q11.23	PPP1R16B: Intron Variant
SNP084	20	20q13.12	WFDC9: Intron Variant
SNP148	20	20q13.2	CYP24A1: Synonymous Variant
SNP190	20	20q13.2	LOC107984001: Intron Variant
SNP194	21	21q21.3	ADAMTS1: 2KB Upstream Variant
SNP073	21	21q22.11	MIS18A: Intron Variant
SNP045	21	21q22.3	PKNOX1: Intron Variant
SNP130	22	22q13.32	None
SNP167	22	22q13.33	None

Abbreviations: SNP, single nucleotide polymorphism
